# Assessing the Utility of Multiplexed Polymerase Chain Reaction in Detecting Microorganisms Causing Infections in Critically ill Patients

**DOI:** 10.1007/s00284-023-03461-3

**Published:** 2023-09-21

**Authors:** Pedro Garrido, Xavier Gabaldó-Barrios, Isabel Pujol-Bajador, Luis Fernández, Frederic Ballester, Raquel Garrido, Pitter Cueto, Jordi Camps, Immaculada Vallverdú

**Affiliations:** 1 Intensive Care Unit, Hospital Universitari de Sant Joan, Salut Sant Joan de Reus-Baix Camp, Av. Dr. Josep Laporte 2, 43204 Reus, Spain; 2 Microbiology Laboratory, Hospital Universitari de Sant Joan, Salut Sant Joan de Reus-Baix Camp, Av. Dr. Josep Laporte 2, 43204 Reus, Spain; 3 Department of Basic Health Sciences, Unit of Microbiology, Faculty of Medicine and Health Sciences, Institut d’Investigació Sanitària Pere Virgili, Universitat Rovira i Virgili, C. Sant Llorenç S/N, 43201 Reus, Spain; 4 Department of Medicine and Surgery Unitat de Recerca Biomèdica, Institut d’Investigació Sanitària Pere Virgili, Hospital Universitari de Sant Joan, Universitat Rovira i Virgili, Salut Sant Joan de Reus-Baix Camp, Av. Dr. Josep Laporte 2, 43204 Reus, Spain

## Abstract

Early sepsis diagnosis is crucial for implementing adequate antibiotic therapy and for patient survival. This study investigated whether using multiplexed PCR for detecting microorganisms in critical septic patients affects initial antibiotic treatment and compared it to microbiological culture. It also explored scenarios where PCR is more effective in clinical practice. One hundred nineteen specimens (83 blood and 36 respiratory specimens) belonging to 93 patients were analyzed. Multiplexed PCR determinations were performed using the FA-BCID Panel (bioMérieux) for blood samples and the FA-Pneumo for respiratory samples. The mean turnaround times were 1.7 h for the FA-BCID and 1.5h for the FA-Pneumo. Conversely, they were 96.1 h for blood cultures and 72.3 h for respiratory cultures. FA-BCID showed a mean sensitivity of 97% and specificity of 100%. FA-Pneumo showed a sensitivity of 100% and specificity of 90%. However, the positive predictive value was only 39%. Discrepancies were common in polymicrobial samples. Based on the PCR results, initial empirical treatment should have been changed in 71% of patients with bloodstream infections and 61% with respiratory infections. We conclude that multiplexed PCR improves the response time in identifying germs with a high degree of coincidence for blood cultures and moderate for respiratory cultures. These results highlight the importance of PCR in choosing an appropriate antibiotic therapy.

## Introduction

Sepsis is a life-threatening condition in which the organism's response to infection leads to organ dysfunction [1]. It is a common reason for Intensive Care Unit (ICU) admission, and critically ill patients are at an increased risk of healthcare-associated infections with high mortality rates [2]. Early diagnosis, life support measures, and antibiotic therapy can improve the prognosis [3]. However, identifying the causative organism can be challenging, as it usually relies on microbiological cultures that may take time. Delays in the prescription of antibiotics can increase mortality in patients with sepsis, but inadequate administration of antibiotics can worsen the outcome [4–6]. Indeed, international guidelines support the initial use of empirical broad-spectrum antibiotics in patients at higher risk of complications or death to avoid treatment failures, potentially leading to unnecessary treatments and associated risks [7–9].

To address these challenges, rapid diagnostic tests that identify the germs causing the infection are crucial for optimal sepsis control [10]. Nucleic acid amplification methods, such as multiplexed polymerase chain reaction (PCR), have shown high diagnostic efficacy [11] and can reduce the time to detect some antibiotic resistance mechanisms [12, 13]. These methods can optimize the initial antibiotic treatment, reducing broad-spectrum antibiotic use and associated risks [14, 15]. However, they have limitations, such as false negatives and positives.

The main objective of this study was to investigate whether introducing the multiplexed PCR technique for early detection of microorganisms in critical septic patients leads to changes in the initial empirical antibiotic therapy recommended by the Intensive Medicine Department guidelines at our hospital. Additionally, we aimed to compare the identification of germs using the multiplexed PCR technique and microbiological culture in blood and respiratory samples from critical septic patients with bacteremia or pneumonia who required mechanical ventilation. We also analyzed the scenarios in which the multiplexed PCR technique may be more effective in clinical practice.

## Methods

### Participants and Study Design

**Fig. 1:**
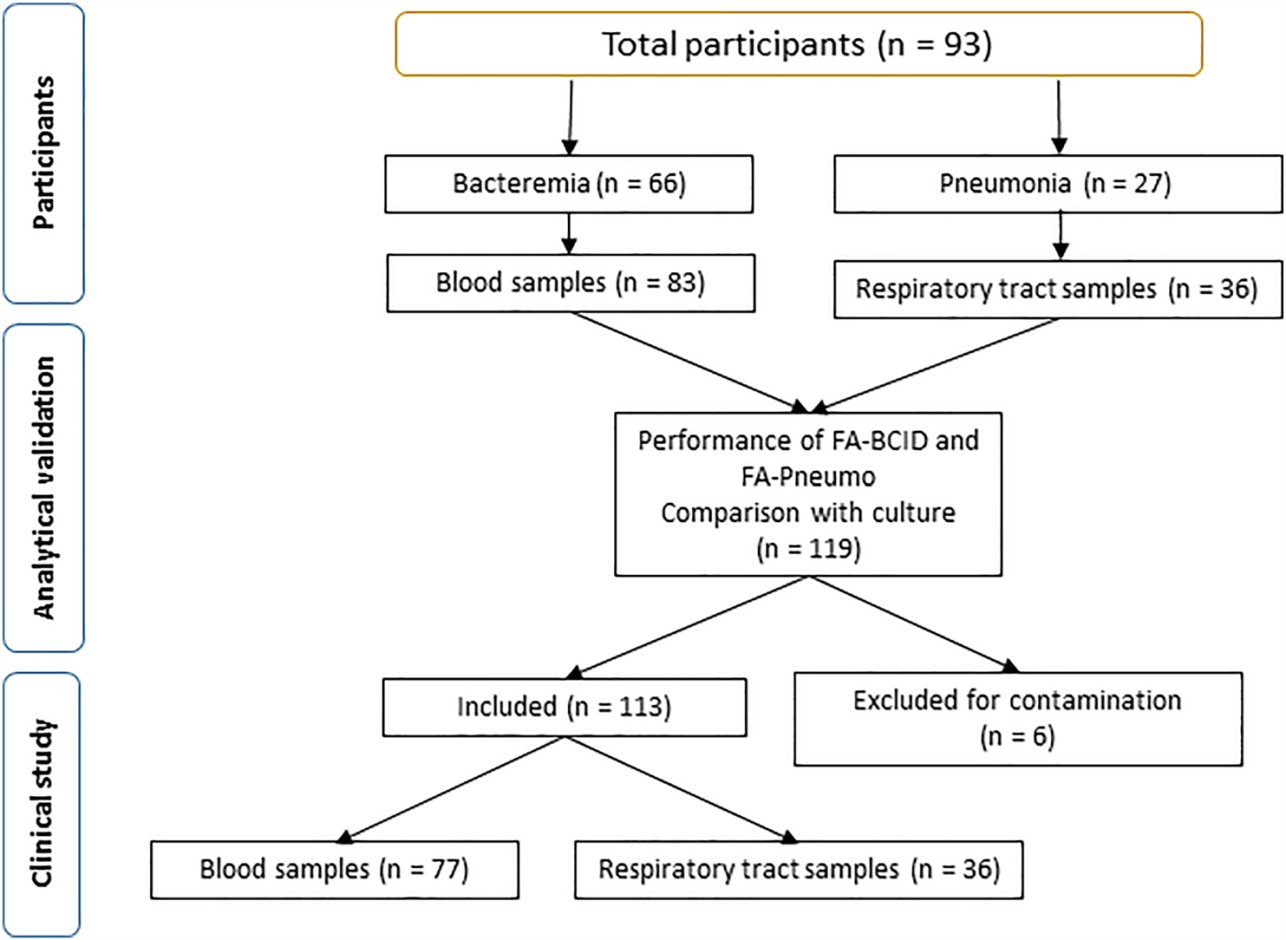
Flowchart detailing the number of patients and samples included in the study

We conducted a retrospective observational study in the medical-surgical ICU of the *Hospital Universitari de Sant Joan de Reus* between January 2021 and July 2022. We included 93 consecutively admitted patients who had bacteremia (*n* = 66) or suspected ventilation-associated pneumonia (VAP, *n* = 27) and underwent diagnostic tests to identify germs using microbiological cultures and multiplexed PCR, per the discretion of the responsible physician. Initially, we conducted an analytical validation study to assess the performance and accuracy of multiplexed PCR methods in 119 samples, comprising 83 blood samples and 36 respiratory tract samples. Some patients provided more than one sample for analysis. Subsequently, our focus shifted to investigating the clinical relevance of the PCR results in patient management. Six blood samples were omitted from the study due to contamination issues, resulting in the final analysis being conducted on 77 blood samples and 36 respiratory tract samples, totaling 113 samples (Fig. 1). Patients with limited therapeutic support were excluded based on medical judgment. The ICU in which this study was conducted only accepts adult patients, since children are cared for in their own pediatric ICU. As a result, all participants were over 18 years of age. VAP was defined as a new or progressive infiltrate on chest radiographic imaging, in addition to two or more indices including fever, purulent respiratory secretions, abnormal routine cell count, or decline in oxygenation, according to the criteria of the Infectious Diseases Society of America [16]. We considered the use of antimicrobials for up to five days before obtaining samples for microbiological cultures and PCR as previous antibiotic therapy. Respiratory samples obtained through tracheal aspirate (TA, *n* = 28) or bronchoalveolar lavage (BAL, *n* = 8) and positive blood cultures showing potentially pathogenic germs during incubation were analyzed by multiplexed PCR and by using conventional methods.

Bacteremia was defined as the presence of potentially pathogenic bacteria in the bloodstream. Fast-growing staphylococci are more frequently associated with true bacteremia. We considered fast-growing staphylococci those detected in less than 8 h by the automated detection system BacT/ALERT 3D (bioMérieux SA, Marcy-L’Étoile, France). In any case, microorganisms of the skin microbiota or ambient air, such as coagulase-negative staphylococci, *Propionibacterium*, *Micrococcus*, *Corynebacterium*, and *Bacillus*, among others, were excluded from the clinical study after a careful analysis of the clinical characteristics of the patient. To diagnose VAP from a microbial point of view, bacterial counts equal to or greater than 10^6^ cfu/mL in TA and equal to or greater than 10^4^ cfu/mL in BAL were used as the cutoff point, following the recommendations of the Spanish Society of Infectious Diseases and Clinical Microbiology [17]. We only accepted respiratory samples from the lower respiratory tract when they met specific criteria, including the presence of leukocytes and the absence of squamous epithelial cells. The samples were carefully examined under a low magnification (100X) microscope, analyzing 20 to 40 fields with more than 25 leukocytes per field and less than ten squamous epithelial cells per field [18]. Lower respiratory tract samples were assessed for the presence of leukocytes and the absence of squamous epithelial cells.

We used a MALDI-TOF VITEK MS System mass spectrometer (bioMérieux) to identify the bacterial strains. This method employs matrix-assisted laser desorption/ionization time-of-flight mass spectrometry technology to identify bacterial strains based on their unique protein profiles. The identification process involves mixing a bacterial strain sample with a matrix solution on a target plate. This plate is then inserted into the VITEK MS instrument, where a laser ionizes the sample molecules, generating a spectrum of ions that are accelerated through a light tube. The time these ions take to reach the detector is measured, producing a mass spectrum or protein profile for the bacterium. This protein profile is subsequently compared against an extensive database containing profiles of known microorganisms, enabling rapid identification of the bacterial strain. Unlike some methods that use a predefined cutoff value, the VITEK MS system provides a "confidence level" for each identification result. The confidence level is categorized as high, medium, or low, represented by different symbols or colors (green, orange, or red, respectively). A high confidence level indicates that the identified protein profile matches with 99.9% concordance to a microorganism listed in the database. In the case of an intermediate level of confidence, the protein profile may correspond to several different organisms (up to three), requiring the expertise of a microbiologist to make a final determination, often supplemented by additional complementary tests. Finally, a low confidence level implies that the system cannot provide any specific identification for the bacterial strain. Additionally, the antibiotic sensitivity of the identified strains was determined using the automated VITEK 2 MS System (bioMérieux) and strip diffusion methods (Etest®, bioMérieux) according to criteria from the European Committee on Antimicrobial Susceptibility Testing (EUCAST) [19]. This method allowed for the determination of which antibiotics would be most effective in treating the infection caused by the identified bacterial strains. The antibiotic sensitivity profile of the identified bacterial strains was assessed by determining the minimum inhibitory concentrations (MIC).

Multiplexed PCR determinations in blood cultures were performed using the BioFire Filmarray Blood Culture Identification Panel (FA-BCID, bioMérieux). This system detects nucleic acids of 27 pathogens commonly associated with bacteremia. We used the BioFire Filmarray Pneumonia Panel (FA-Pneumo, bioMérieux) for respiratory samples. This system targets 24 pathogens and 10 antibiotic resistance profiles, comprehensively analyzing the bacterial strains in the respiratory samples, including any antibiotic-resistant strains. The bacterial loads obtained from these tests were expressed in the number of copies/mL, ranging from 10^4^ to > 10^7^ copies/mL.

In patients with suspected bacteremia, the administration of antibiotics was usually initiated empirically before or simultaneously with the FA-BCID test, and prior to the confirmation by blood cultures. Similarly, antibiotic treatment may have been initiated in patients with suspected pneumonia before the concurrent collection of microbiological culture and FA-Pneumo samples. The attending physician subsequently determined whether to discontinue or modify ongoing antibiotic therapy or to initiate empirical treatment based on the updated protocol of the hospital's ICU following receipt of the PCR test results. After receiving the final report of the microbiological culture and corresponding antibiogram, the attending physician made appropriate adjustments to the antibiotic therapy.

### Data Analyses

We compared the average time of results between the molecular method and conventional microbiological culture, which included antibiotic resistance testing. We assessed the sensitivity, specificity, positive (PPV) and negative predictive values (NPV), and κ index of the FA-BCID and FA-Pneumo tests by using the bacterial culture as the gold standard.

For respiratory samples, we compared FA-Pneumo with the microbiological culture to classify the results as completely concordant (when both tests detect the same pathogens or lack thereof), partially concordant (when both tests detect the same pathogen, but one detects an additional pathogen not found in the other test), or non-concordant (when the pathogen detected by one test differs from the other). Our team, composed of experts in critical patient infections and microbiologists analyzed each case individually, along with analytical, radiographic, and microbiological results. We evaluated whether empirical or PCR-guided antibiotic therapy was appropriate based on the pathogens isolated in microbiological cultures, according to the antibiogram. We assessed any changes in the antimicrobial treatment regimen after knowing the PCR test result. Antibiotic therapy modifications were classified as de-escalation (substitution by an adequate antibiotic with a smaller spectrum), initiation, withdrawal, or escalation (introduction of an effective antibiotic against bacteria causing an infection that was not adequately treated before knowing the results of molecular detection), using criteria established by Weiss et al. [20].

We assessed the diagnostic efficacy of procalcitonin, C-reactive protein, leukocytes, neutrophils, lymphocytes, D-dimer, and lactate as biomarkers of inflammation in VAP by measuring their levels using pre-established thresholds indicative of potentially pathogenic microbial growth. Patient data were extracted from medical records and anonymized before statistical analysis using Microsoft Excel (Microsoft, Redmond, WA, USA) software. We used GraphPad Prism 9 (Dotmatics Bishop's Stortford, UK) for all analyses. Categorical variables were shown as absolute numbers and percentages and compared by the χ-square test. Continuous variables were shown as medians and interquartile ranges and compared by the Mann–Whitney U test.

### Biochemical and Hematological Analyses

We measured serum procalcitonin concentrations by an Elecsys cobas® e411 analyzer (Hoffmann-La Roche, Basel, Switzerland), C-reactive protein by a cobas® 8000 modular analyzer (Hoffman-La Roche), plasma lactate, with a RAPIDPoint 500 system analyzer (Siemens Healthlineers, Erlangen, Germany), and hematological parameters by a Sysmex XN analyzer (Sysmex Corporation, Kobe, Japan).

### Ethical Aspects

The study was approved by the *Comitè d'Ètica i Investigació en Medicaments* (Institutional Review Board) of the *Institut d'Investigació Sanitària Pere Virgili* (Ref. 180/2022). The requirement to obtain written informed consent from patients was waived.

## Results

### Demographic Characteristics

The mean age of the patient cohort was 65 years, with a majority of 73.9% being male. Among the patients subjected to blood sample analysis, the most prevailing comorbidities were urinary tract infection, ventilator-associated pneumonia, and central line-associated bloodstream infection. Conversely, coronavirus infection emerged as the dominant condition in patients whose respiratory tract samples were analyzed (Table 1).

**Table 1:** Demographic features and clinical characteristics

Characteristic	FA-BCID (*n* = 66)	FA-Pneumo (*n* = 27)	All patients (*n* = 93)
Age (years)	66 (58–74)	63 (49–70)	65 (54–73)
Sex			
Men	47 (71.2)	21 (80.8)	68 (73.9)
Women	19 (28.8)	5 (19.2)	24 (26.1)
SAPS II	33.0 (26.8–47.8)	30.0 (16.5–38.0)	33.0 (25.0–45.0)
APACHE II	15.0 (10.0–19.0)	13.0 (7.8–18.0)	15.0 (9.3–18.0)
Underlying disease			
Coronavirus	0	20 (74.1)	20 (21.5)
Urinary tract infection	13 (19.47)	0	13 (14.0)
Ventilator-associated pneumonia (VAP)	12 (18.2)	0	12 (13)
Central line-associated bloodstream infection	10 (15.2)	0	10 (12.9)
Pneumonia	5 (7.6)	2 (7.4)	7 (7.5)
Primary bacteriemia	6 (9.1)	0	6 (6.5)
Hospital-acquired infection	4 (6.1)	0	4 (4.3)
Heart failure	2 (3.0)*	2 (7.4)*	4 (4.3)
Pneumonia in an immunosuppressed patient	2 (3.0)	1 (3.7)	3 (3.1)
Cholangitis	3 (4.5)	0	3 (3.1)
Cholecystitis	2 (3.0)	0	3 (3.1)
Endocarditis	2 (3.0)	0	2 (2.1)
Hypoxemic respiratory failure	0	2 (7.4)	2 (2.1)
Peritonitis	1 (1.5)	0	1 (1.0)
Oral abscess	1 (1.5)	0	1 (1.0)
Early nasal intermittent positive pressure ventilation (NIPPV)	1 (1.5)	0	1 (1.0)
Meningitis	1 (1.5)	0	1 (1.0)
Osteomyelitis	1 (1.5)	0	1 (1.0)

^*^FA-BCID Filmarray and FA-Pneumo Filmarrays were performed in the same patient

### Analytical Validation: Turnaround Time and Pathogen Detection

The mean turnaround time for the FA-BCID panel was 1.7 h (min–max: 1.1–5.0 h), and that of the FA-Pneumo panel was 1.5 h (min–max: 0.7–3.3 h). The mean turnaround time for conventional microbiological methods was 96.1 h (min–max: 24.6–172.6 h) for blood cultures and 72.3 h (min–max: 22.1–187.7 h) for cultures of respiratory samples.

The performance of FA-BCID and FA-Pneumo panels concerning each pathogen is shown in Tables 2 and 3, respectively. FA-BCID showed a sensitivity of 97% (95% confidence interval 90–99%) and specificity of 100% (95% confidence interval 99–100%). The sensitivity for each of the pathogens was 100% except for *Staphylococcus aureus* (85%, 2/13 cases detected only by culture) and *Escherichia coli* (96%, 1/23 patients seen only by culture). The most prevalent pathogens were *E. coli* (23 patients), *Pseudomonas aeruginosa* (11 cases), *S. aureus* (13 patients), and Coagulase-Negative *Staphylococcus* (9 patients). The FA-Pneumo detected at least one pathogen in 31 of the 36 tested specimens, yielding a positivity rate of 86.1%. This panel showed a sensitivity of 100% (95% confidence interval 86–100%) and specificity of 90% (95% confidence interval 89–94%) but an overall PPV of 39% (95% confidence interval 29–51%). For example, all cases of *Enterobacter cloacae* were detected by FA-Pneumo but not by culture methods (*n* = 6). The most prevalent bacteria were *S. aureus* (*n* = 13 on both FA-Pneumo and culture), followed by *Haemophilus influenzae* (*n* = 4 on both FA-Pneumo and culture and *n* = 3 only by FA-Pneumo), *E. coli* (*n* = 5 on both FA-Pneumo and culture and *n* = 2 only by FA-Pneumo) and *P. aeruginosa* (*n* = 6 on both FA-Pneumo and culture). No microorganism present in the panel was detected only by culture.

**Table 2:** Performance of the FA-BCID panel relative to conventional culture*

Bacterial pathogen	FA-BCID+ and culture+	FA-BCID+ and culture−	FA-BCID− and culture+	FA-BCID− and culture−	Sensitivity (95% CI)	Specificity (95% CI)	PPV (95% CI)	NPV (95% CI)	Kappa coefficient
*Enterococcus *sp	7	0	0	76	1.00 (0.56–1.00)	1.00 (0.94–1.00)	1.00 (0.56–1.00)	1.00 (0.94–1.00)	1.00
*Listeria Monocystogenes*	0	0	0	83		1.00 (0.94–1.00)	–	1.00 (0.94–1.00)	–
*Coagulase-Negative Staphylococcus (CNS)*	9	0	0	74	1.00 (0.63–1.00)	1.00 (0.94–1.00)	1.00 (0.63–1.00)	1.00 (0.94–1.00)	1.00
*Staphylococcus aureus*	11	0	2	70	0.85 (0.54–0.97)	1.00 (0.94–1.00)	1.00 (0.68–1.00)	0.97 (0.89–1.00)	0.90
*Streptococcus *sp	4	0	0	79	1.00 (0.40–1.00)	1.00 (0.94–1.00)	1.00 (0.40–1.00)	1.00 (0.94–1.00)	1.00
*Streptococcus agalactiae*	0	0	0	83	–	1.00 (0.94–1.00)	–	1.00 (0.94–1.00)	–
*Streptococcus pyogenes*	0	0	0	83	–	1.00 (0.94–1.00)	–	1.00 (0.94–1.00)	–
*Streptococcus pneumoniae*	1	0	0	82	1.00 (0.05–1.00)	1.00 (0.94–1.00)	1.00 (0.05–1.00)	1.00 (0.94–1.00)	1.00
*Acinetobacter baumanii*	0	0	0	83	–	1.00 (0.94–1.00)	–	1.00 (0.94–1.00)	–
*Haemophilus influenzae*	0	0	0	83	–	1.00 (0.94–1.00)	–	1.00 (0.94–1.00)	–
*Neisseria meningitiditis*	0	0	0	83	–	1.00 (0.94–1.00)	–	1.00 (0.94–1.00)	–
*Pseudomonas aeruginosa*	11	0	0	72	1.00 (0.68–1.00)	1.00 (0.94–1.00)	1.00 (0.68–1.00)	1.00 (0.94–1.00)	1.00
*Enterobacter cloacae*	0	0	0	83	–	1.00 (0.94–1.00)	–	1.00 (0.94–1.00)	–
*Escherichia coli*	22	0	1	60	0.96 (0.76–0.99)	1.00 (0.93–1.00)	1.00 (0.82–1.00)	0.98 (0.90–1.00)	0.97
*Klebsiella oxytoca*	2	0	0	81	1.00 (0.2–1.00)	1.00 (0.94–1.00)	1.00 (0.2–1.00)	1.00 (0.94–1.00)	1.00
*Klebsiella pneumoniae*	8	0	0	75	1.00 (0.6–1.00)	1.00 (0.94–1.00)	1.00 (0.6–1.00)	1.00 (0.94–1.00)	1.00
*Proteus sp*	2	0	0	81	1.00 (0.2–1.00)	1.00 (0.94–1.00)	1.00 (0.2–1.00)	1.00 (0.94–1.00)	1.00
*Serratia marcescens*	4	0	0	79	1.00 (0.40–1.00)	1.00 (0.94–1.00)	1.00 (0.40–1.00)	1.00 (0.94–1.00)	1.00
*Candida albicans*	2	0	0	81	1.00 (0.2–1.00)	1.00 (0.94–1.00)	1.00 (0.2–1.00)	1.00 (0.94–1.00)	1.00
*Candida glabrata*	1	0	0	82	1.00 (0.05–1.00)	1.00 (0.94–1.00)	1.00 (0.05–1.00)	1.00 (0.94–1.00)	1.00
*Candida krusei*	0	0	0	83	–	1.00 (0.94–1.00)	–	1.00 (0.94–1.00)	–
*Candida parapsilosis*	0	0	0	83	–	1.00 (0.94–1.00)	–	1.00 (0.94–1.00)	–
*Candida tropicalis*	0	0	0	83	–	1.00 (0.94–1.00)	–	1.00 (0.94–1.00)	–
Total	84	0	3	1822	0.97 (0.90–0.99)	1.00 (0.99–1.00)	1.00 (0.95–1.00)	1.00 (0.99–1.00)	0.98

^*^Five cases with pathogen growth in the blood culture were not considered as there was not a specific target in the Filmarray. The microorganisms detected were: *Prevotella buccae, Clostridium perfringens, Prevotella bivia, Moraxella catarrhalis, and Phocaeicola vulgatus*. Sensitivity, specificity, PPV, and NPV, were calculated by comparing the results for conventional culture with those of FilmArray only for bacterial pathogens present in the molecular panel. Performance was measured considering bacterial culture as the gold standard reference method

*FA*-*BCID* BioFire Filmarray Blood Culture Identification Panel; *NPV* Negative predictive value; *PPV* Positive predictive value

**Table 3:** Performance of the FA-Pneumo panel relative to conventional culture*

Bacterial pathogen		FA-Pneumo+ and culture+		FA-Pneumo+ & culture−	FA-Pneumo− and culture+	FA-Pneumo− and culture−	Sensitivity (95%CI)	Specificity (95%CI)	PPV (95%CI)	NPV (95%CI)	Kappa coefficient
*Acinetobacter calcoaceticus-baumanii complex*		1		0	0	35	1.00 (0.05–1.00)	1.00 (0.88–1.00)	1.00 (0.05–1.00)	1.00 (0.88–1.00)	1.00
*Enterobacter cloacae*		0	6		0	30		0.83 (0.67–0.93)	0.00 (0.02–0.48)	1.00 (0.86–1.00)	0.00
*Escherichia coli*		5	2		0	29	1.00 (0.46–1.00)	0.94 (0.81–0.99)	0.71 (0.30–0.95)	1.00 (0.85–1.00)	0.80
*Haemophilus influenzae*		4	3		0	29	1.00 (0.40–1.00)	0.91 (0.74–0.98)	0.57 (0.20–0.88)	1.00 (0.85–1.00)	0.68
*Klebsiella aerogenes*		0	0		0	36	–	1.00 (0.88–1.00)	–	1.00 (0.88–1.00)	–
*Klebsiella oxytoca*		0	0		0	36	–	1.00 (0.88–1.00)	–	1.00 (0.88–1.00)	–
*Klebsiella pneumoniae group*		1	0		0	35	1.00 (0.05–1.00)	1.00 (0.88–1.00)	1.00 (0.05–1.00)	1.00 (0.88–1.00)	1.00
*Moraxella catarrhalis*		0	1		0	35	–	0.97 (0.84–1.00)	–	1.00 (0.88–1.00)	0.00
*Proteus spp.*		0	2		0	34	–	0.94 (0.80–0.99)	–	1.00 (0.87–1.00)	0.00
*Pseudomonas aeruginosa*		6	0		0	30	1.00 (0.52–0.98)	1.00 (0.86–1.00)	1.00 (0.52–0.98)	1.00 (0.86–1.00)	1.00
*Serratia marcescens*		0	0		0	36	–	1.00 (0.88–1.00)	–	1.00 (0.88–1.00)	–
*Staphylococcus aureus*		13	0		0	23	1.00 (0.72–1.00)	1.00 (0.82–1.00)	1.00 (0.72–1.00)	1.00 (0.82–1.00)	1.00
*Streptococcus agalactiae*	1		2		0	33	1.00 (0.05–1.00)	0.94 (0.79–0.99)	0.33 (0.02–0.87)	1.00 (0.87–1.00)	0.48
*Streptococcus pneumoniae*		0	1		0	35	–	0.97 (0.84–1.00)	–	1.00 (0.88–1.00)	0.00
*Streptococcus pyogenes*		0	0		0	36	–	1.00 (0.88–1.00)	–	1.00 (0.88–1.00)	–
*Legionella pneumophila*		0	0		0	36	–	1.00 (0.88–1.00)	–	1.00 (0.88–1.00)	–
Total		31	48		0	528	1.00 (0.86–1.00)	0.92 (0.89–0.94)	0.39 (0.29–0.51)	1.00 (0.99–1.00)	0.53

^*^Five cases with pathogen growth in the culture were not considered as there was not a specific target in the Filmarray. The microorganisms detected were *Candida albicans* (2), *Burkholderia cepacia, Klebsiella aerogenes and Morganella morganii*. Sensitivity, specificity, PPV, and NPV, were calculated by comparing the results for conventional culture with those of FilmArray only for bacterial pathogens present in the molecular panel. Performance was measured considering bacterial culture as the gold standard reference method

*FA*-*Pneumo* BioFire Filmarray Pneumonia Panel; *NPV* Negative predictive panel; *PPV* Positive predictive panel

Discrepancies between FA-Pneumo and culture results are shown in more detail in Table 4. These methods yielded a non-concordant, partially concordant, and completely concordant result in 13.9% (5/36), 30.5% (11/36), and 55.6% (20/36) of the analyzed samples, respectively. Polymicrobial samples were those with a higher degree of discrepancy, being mostly non-concordant or partially concordant (*P* < 0.001). We observed no significant differences regarding the sample type (TA or BAL) or pathogen load.

**Table 4:** Comparison between the FA-Pneumo and the culture according to the results of the FA-Pneumo, the type of respiratory sample and the bacterial load provided by the FA-Pneumo

	Total samples*	Concordance between the FA-Pneumo and the culture			*P*-value
		Non-concordant	Partially concordant	Completely concordant	
Results of FA-Pneumo					
No microbes	5	0		5	< 0.001
Monomicrobial	11	2		9	
Polymicrobial	20	4	10	6	
Type of Sample					
Tracheal aspirate	28	5	8	15	0.894
Bronchoalveolar lavage fluid	8	1	2	5	
Bacterial loads of the FA-Pneumo					
≤ 10^5^ copies/mL	24	6	10	8	0.240
> 10^5^ copies/mL	39	10	26	27	

*In the row of bacterial loads, as some samples are polymicrobial, the total number of cases analyzed (63) exceeds the number of specimens (36). The number of microorganisms detected is 58 by the two methods plus five seen by culture but which are not part of the panel (*Staphylococcus epidermidis* = 1, *Burkholderia cepacia* = 1, *Candida albicans* = 2, *Morganella morganii* = 1). FA-Pneumo: BioFire Filmarray Pneumonia Panel. We evaluated the possibility of polymicrobial pneumonia diagnosis when the bacterial counts from tracheal aspirate and bronchoalveolar lavage samples for each isolated microorganism met prescribed criteria [16]. If the values fell below the specified thresholds, they were deemed as contamination, regardless of any concurrence with PCR results

Most cases (about 42%) detected by culture in the presence of a positive FA-Pneumo panel were characterized by high bacterial loads (> 10^7^ copies/ mL) (Table 5). Six cases (11.5%) not detected by the FA-Pneumo panel showed a microbial culture with bacterial loads ranging between 10^4^ and 10^6^ cfu/mL (Table 5).

**Table 5:** Concordance of bacterial loads between culture and FA-Pneumo panel. Only bacterial pathogens present in the molecular panel were considered

		FA-Pneumo (copies/mL)				
		> 10^7^/10^7^	10^6^	10^5^	10^4^	Not detected
Culture (cfu/mL)	> 10^6^	17	0	0	0	3
	10^5^	2	1	0	0	2
	10^4^	2	2	0	0	1
	10^3^	1	0	0	0	0
	Not detected	6	6	2	8	0

FA-Pneumo: BioFire Filmarray Pneumonia Panel

The FA-BCID test detected eight cases with the antibiotic resistance gene *mecA/C* and two with *blaCTX-M*, agreeing with the antibiotic susceptibility testing results (Table 6). FA-Pneumo identified no antimicrobial resistance gene.

**Table 6:** List of resistance genes detected by the FA-BCID panel and conventional microbiological methods

Bacterial culture	FA-BCID results		Susceptibility testing results*						Initial antibiotic therapy	Microbiological evaluation
Strain	Strain	Gene	OXA	CAZ	CTX	FEP	ETP	IPM		
*S. epidermidis*	*S. epidermidis*	*Mec*_*A/C*_							MEM	Contamination
*E. coli*	*E. coli*	*Bla*_*CTX-M*_		R (4)	R (≥ 64)	R (2)	S (≤ 0.50)	S (≤ 0.25)	MEM	Bacteremia
*S. epidermidis*	*S. epidermidis*	*Mec*_*A/C*_	R (≥ 4)						LZD	Bacteremia
*S. epidermidis*	*S. epidermidis*	*Mec*_*A/C*_							LZD	Contamination
*S. epidermidis*	*S. epidermidis*	*Mec*_*A/C*_	R (≥ 4)						PIP/TAZ	Bacteremia
*S. epidermidis*	*S. epidermidis*	*Mec*_*A/C*_							–	Contamination
*S. epidermidis*	*S. epidermidis*	*Mec*_*A/C*_	R (≥ 4)						–	Contamination
*S. epidermidis*	*S. epidermidis*	*Mec*_*A/C*_	R (≥ 4)						–	Bacteremia
*S. epidermidis*	*S. epidermidis*	*Mec*_*A/C*_	R (≥ 4)						AMP,CRO, CLO	Bacteremia
*E. coli*	*E. coli*	*Bla*_*CTX-M*_		I (4)	R (≥ 64)	I (2)	S (≤ 0.50)	S (≤ 0.25)	MEM	Bacteremia

*Results are shown following the European Committee on Antimicrobial Susceptibility Testing recommendations. For example, R (4) means resistant to antibiotic with a minimum inhibitory concentration of 4 mg/L, I (4) means susceptible at increased exposure of 4 mg/L, and S (<0.25) means sensitive to antibiotic with a minimum inhibitory concentration <0.25 mg/L. Some coagulase-negative staphylococci were considered clinically significant (catheter bacteremia) or contaminants according to the review by the experts in critical patient infections and the microbiologists

*AMP* Ampicillin; *BlaCTX-M* Extended spectrum beta-lactamase (cefotaximase); *CAZ* Ceftazidime; *CLO* Clotrimazole; *CRO* Ceftriaxone; *CTX* Cefotaxime; *ETP* Ertapenem; *FEP *Cefepime; *IPM* Imipenem; *LZD* Linezolid; *MecA*/*C* Gene A or C that produces a mutated penicillin binding protein coded for methicillin resistance; *MEM* Meropenem; *OXA* Oxacillin; *PIP* Piperacillin/tazobactam

### Clinical Study: The Potential Impact of FA-BCID and FA-Pneumo on Treatment

Based on the results of FA-BCID, initial empirical treatment should have been changed in 55/77 (71.4%) of these cases, with antibiotic de-escalation in 42/55 (76.4%) and antibiotic escalation in 13/55 (23.6%) (Table 7).

**Table 7:** General results of the Fsilmarrays and impact on antibiotic therapy^1^

Results of FilmArray		Previous antibiotic treatment^2^			On antibiotics when tested			Antibiotic modification			Initial empirical treatment may have been changed?			Type of antibiotic modification			
		Yes	No	*P*-value	Yes	No	*P*-value	Yes	No	*P****-***value	Yes	No	*P*-value	De-escalation	Escalation	Stop	*P*-value
Bloodstream																	
Negative	n = 1	1	0	0.664	–	–	–	0	1	0.577	0	1	0.243	–	–	–	0.765
Monomicrobial	n = 61	58	3		–	–		10	51		45	16		34	11	–	
Polymicrobial	n = 15	15	0		–	–		1	14		10	5		8	2	–	
All	n = 77	74	3		–	–		11	66		55	22		42	13	–	
Pneumonia																	
Negative	n = 5	2	3	0.075	3	2	0.026	1	4	0.266	3	2	0.625	0	0	3	0.012
Monomicrobial	n = 11	1	10		9	2		0	11		8	3		5	2	1	
Polymicrobial	n = 20	10	10		20	0		1	19		11	9		9	0	2	
All	n = 36	13	23		32	4		2	34		22	14		14	2	6	

^1^Patients in which a single microorganism evaluated as blood culture contaminant was detected are not considered

^2^Antibiotic treatment received up to 5 days prior to the performance of Filmarray

A total of 36 cases had clinically suspected pneumonia, according to the retrospective chart review. Most patients (88.9%) received an antibiotic when the FA-Pneumo was performed. Only 2 cases had their treatment modified, but it should have to be done in most patients: 14 (63.6%) with antibiotic de-escalation, 2 (9.1%) with antibiotic escalation, and 6 (27.3%) with antibiotic stop (Table 7).

### Biochemical Parameters in Respiratory Infections for the Detection of a Clinically Relevant Bacterial Infection

**Fig. 2:**
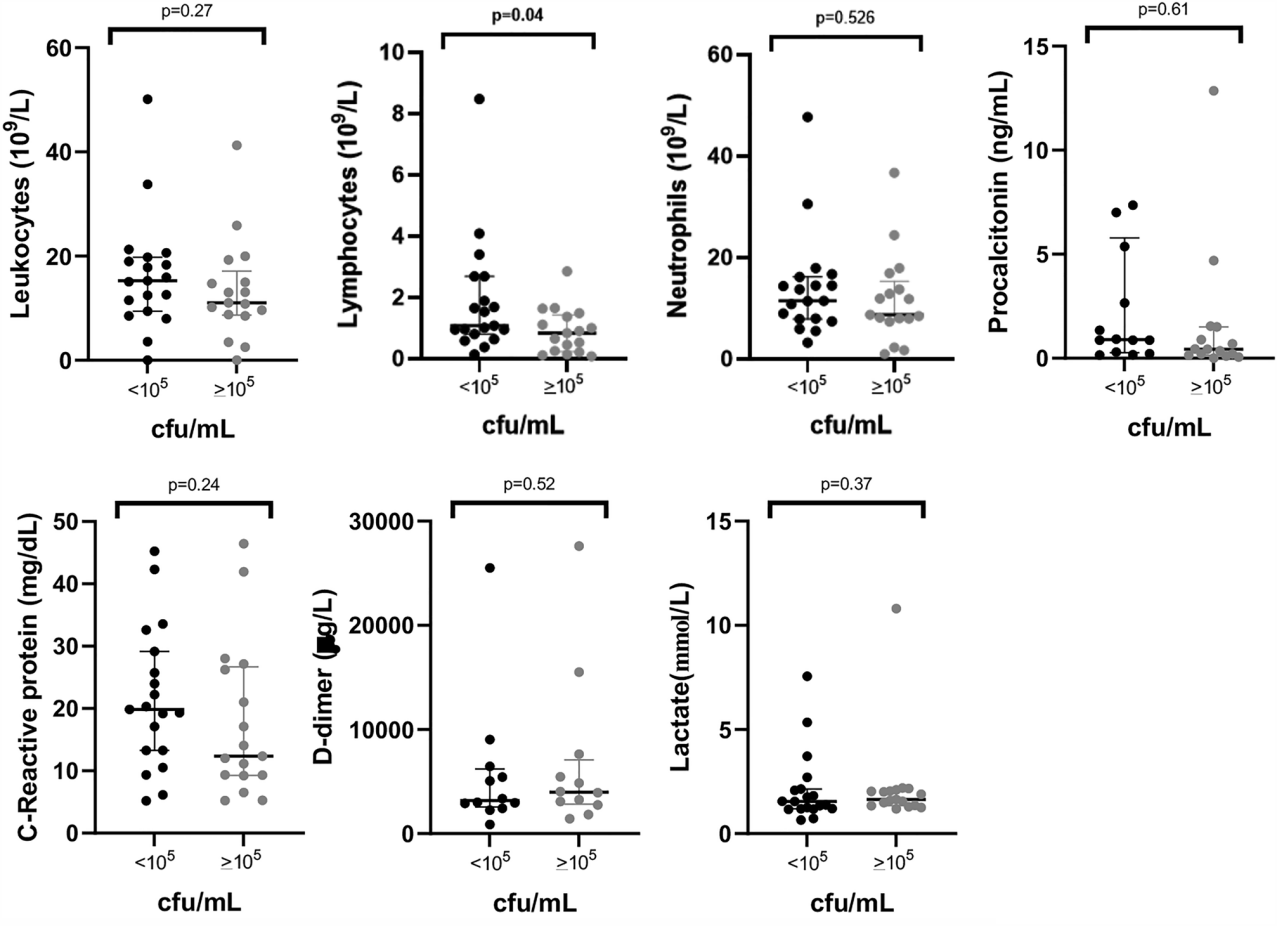
Concentrations of leukocytes, lymphocytes, neutrophils, procalcitonin, C-reactive protein, D-dimer, and lactate according to the bacterial load detected in conventional culture of respiratory samples from patients with suspected ventilation-associated pneumonia (cut-off: 10^5^ cfu/mL)

Finally, we wanted to know if, in patients with pneumonia, some analytical parameters could help us to predict significant bacterial load in culture. However, we could only note that a lower number of lymphocytes correlated with a bacterial load > 10^5^ cfu/mL (*P* = 0.04) (Fig. 2).

## Discussion

The current investigation examines the application of multiplexed PCR-based panels for the early detection of microorganisms in critically ill septic patients in the ICU of *Hospital Universitari de Sant Joan de Reus*. We studied 93 subjects in whom 83 molecular panels of FA-BCID and 36 panels of FA-Pneumo were taken in blood and respiratory culture samples respectively. To assess the diagnostic performance of these methods, we compared them with traditional microbiological cultures. Additionally, we analyzed the impact of these results on clinical outcomes, particularly concerning antibiotic therapy, and we discuss its applicability in daily clinical practice.

Our study shows that, as expected, PCR provided results much faster than microbiological culture and antibiogram analysis. This faster detection time is particularly significant given that the microbiology laboratory at our hospital operates during restricted hours and only on working days. Despite these time limitations, PCR was available without any restrictions, with an average delay of fewer than 2 h from test completion to receiving results due to laboratory programming settings. Therefore, the time to obtain results was considerably shortened, faster than studies conducted with other PCR platforms [12, 21] but comparable to that obtained by other authors in patients with bacteremia [13] or respiratory infections [22] working with the same PCR manufacturer. In some cases, the response time was lengthened, reaching up to 5h for the FA-BCID panel and 3.3h for the FA-Pneumo panel, but this can be explained because our study was carried out in the challenging context of the COVID-19 pandemic, with an abnormally high workload in the Microbiology Laboratory.

We found a high degree of concordance in microorganism classification when comparing samples analyzed by PCR and their corresponding microbiological culture in blood cultures (*k* = 0.97), which is moderate in the case of respiratory samples (*k* = 0.53). Both panels' diagnostic sensitivities and specificities were satisfactory, with diagnostic yields similar to those obtained by other studies despite the use of different marketed PCR platforms [23]. In our research, NPV and PPV in blood cultures by FA-BCDI were 100% on the targets analyzed in the multiplexed PCR panel.

In respiratory samples from patients with pneumonia, our results also show degrees of agreement similar to those published by other authors [21, 24, 25]. Several studies demonstrated high NPV close to 100% in respiratory samples analyzed by PCR [26–28] as we do in respiratory samples where NPV was 100%. However, a PPV of 39% was obtained, lower than that reported in the studies cited above.

Among the most prevalent bacteria detected in respiratory samples by both methods are *S.aureus*, *P.aeruginosa*, and enterobacteria, a common finding in other studies [22, 25–31]. We highlight the absence of growth by the traditional culture of all cases (*n* = 6) of *E. cloacae* detected by FA-Pneumo. All these samples were polymicrobial; in five of six cases, the patients had received antibiotics in the five days before sampling, and in four of six cases, the bacterial load was equal to or less than 10^5^ copies/mL, perhaps due to the previous administration of antibiotics or the competition between microorganisms for nutrients in the culture media, as it was previously reported [32, 33]. We observed that in polymicrobial samples, the degree of agreement was lower, finding a partial correlation in the detection of germs by FA-Pneumo and microbiological culture on ten occasions, seven of which belonged to patients who had received previous antibiotic therapy, an observation already reported by other authors [11, 28, 31, 34].

Despite the lack of consensus regarding specific microbiological load values detected by PCR that can differentiate between infection and colonization, higher loads have been associated with microbial growth in cultures, making it a valid criterion for diagnosis [35]. For instance, loads equal to or exceeding 10^5^ copies/mL are correlated with bacterial growth in microbiological cultures, while loads lower than 10^5^ copies/mL are associated with the absence of microbes [27, 31, 34]. Our observations revealed a strong correlation at 10^7^ copies/mL.

In 5 out of 36 respiratory samples examined, we did not detect any germs in either FA-Pneumo or microbiological cultures, thus reasonably excluding the possibility of bacterial infection. This observation raises doubts about the necessity of initiating empirical antibiotic therapy in such cases. Conversely, we detected negative FA-BCID in five blood culture samples from patients exhibiting clear signs of sepsis due to bacteremia caused by anaerobic bacteria, which are not part of the diagnostic panel by PCR. Hence, knowing the microbiological targets and resistance genes detectable by PCR is crucial. Therefore, the decision to initiate antibiotic therapy in the absence of microbial detection by PCR must be individualized, and an active antibiotic therapy optimization program should be implemented, which is currently absent in our case.

Almost all the blood samples (75/77) came from patients with bacteremia who received antimicrobials while FA-BCID measurement was performed. In most cases, the regimen was kept the same after its realization (66/77, 85.7%), being maintained until microbiological culture and antibiogram results were obtained. The adjustments made (11/77, 14.3%) were due to a lack of adequacy of treatment (identified germs not covered) on five occasions and by de-escalation on six occasions. We dismissed those detections of coagulase-negative *Staphylococcus* in PCR of blood cultures that we considered contaminations according to the clinical context of each patient. The subsequent expert review concluded that in 55/77 cases (71.4%), empirical antibiotic therapy could have been modified, the majority (42/55, 76.36%) in the form of de-escalation. Rule et al. [13] reported antibiotic modification rates in 32% of their patients with bacteremia, being mainly escalated. However, unlike us, a high incidence of multidrug-resistant germs in their series should be noted.

Our study's analysis of respiratory samples revealed that 8 out of 36 patients had already started antibiotic therapy before PCR, 24 out of 36 started antibiotic treatment when performing FA-Pneumo, and four did not receive antibiotic therapy. Only two patients had a modification of their antibiotic regimen after receiving the results of FA-Pneumo. Upon subsequent analysis, we found that in 22 out of 36 cases (61.1%), the antibiotic treatment could have been optimized, mainly through de-escalation (63.6%) and withdrawal (27.3%), and only in 2 out of 22 cases (9%) as escalation, considering the use of beta-lactams as the primary treatment for the infection.

Similar studies conducted on critical patients with suspected respiratory infections have observed comparable results, where modifications in antibiotic therapy were necessary in a significant proportion of cases [11, 24, 29–31, 34].

Seventy-four point one percent of our patients with suspected VAP were admitted to the ICU due to respiratory infection by SARS-CoV-2, so it is worth mentioning a prospective study in critical COVID-19 patients with VAP in which the antibiotic was only withdrawn in one-third of patients with negative PCR [22]. The possible lack of confidence in the PCR technique, which may justify the clinician's decision not to modify the antimicrobial treatment initiated, and the absence of an active antibiotic optimization program prevent obtaining better performance from this test [36].

However, there are challenges in interpreting PCR results in bronchial secretions of critical patients with suspected pneumonia, as there is a possibility of overdiagnosis due to non-viable, contaminating, or colonizing germs that are not responsible for the infection. Hence, the performance and impact on clinical decisions of PCR-based rapid diagnostic tests in serious situations have yet to be established, as they do not necessarily rule out the possibility of infection and therefore, providing empirical antibiotic coverage for potential pathogens would be justifiable. Nonetheless, these tests can still provide valuable help in adjusting early and adequate initial antibiotic therapy in critical patients, especially in environments with a high incidence of infection by multidrug-resistant germs.

We found a low incidence of genetic resistance by PCR, which we confirmed by culture. The absence of multidrug resistance may have contributed to the high rates of adequate empirical coverage in our series, with 90.3% coverage in respiratory samples and 88.4% in blood culture samples. This finding suggests that therapeutic adjustments in the form of escalation were only needed sometimes, as they would be if there were higher rates of resistance.

Next, we investigated whether additional biochemical tests could help predict pneumonia. Lymphopenia was the only parameter correlated with a high bacterial load. Lymphopenia has recently been studied in SARS-CoV-2 infection and is a frequent finding in these patients [37], associated with a worse prognosis [38], and identified as a risk factor for secondary infections [39].

The study has several limitations, primarily due to its retrospective design. It did not involve a team with expertise in interpreting PCR results to offer active support for optimizing antibiotic use. Consequently, decisions to prescribe antibiotics were left to the discretion of the responsible physician. Another limitation was the study's confinement to a single ICU, with a relatively small number of patients and a population of respiratory samples mainly from people affected by COVID-19, with limited participation from other pathologies. Additionally, the low incidence of infections caused by multidrug-resistant organisms reinforces the confidence in the effectiveness of initial empirical treatment in most cases.

We conclude that multiplexed PCR improves the response time in the identification of germs with a high degree of coincidence with respect to blood cultures and moderate in relation to cultures of respiratory samples. These results highlight the importance of PCR in choosing an appropriate antibiotic therapy.

## Data Availability

Data are available from the corresponding author on reasonable request.
